# Righteousness in the Land of Forgetfulness

**DOI:** 10.1007/s10943-013-9813-z

**Published:** 2014-01-30

**Authors:** Mairi R. Finlay

**Affiliations:** Aberdeen, Scotland, UK

**Keywords:** Dementia, Spirituality, Christianity, Personhood

## Abstract

The experience of dementia is raises many important questions about the nature of self and personhood. No disease is experienced in isolation and dementia embodies this. Ideas of loss of self and loss of life feature strongly in dementia and have the potential to profoundly affect a person’s spirituality. The Christian faith offers the possibility of retaining and recovering the sense of personhood and connection with God and others. This allows for the possibility of hope.

## Righteousness in the Land of Forgetfulness

Dementia can be defined as “a chronic or persistent disorder of behaviour and higher intellectual function due to organic brain disease” (Oxford Concise Medical Dictionary 2003). However, this brief medical statement appears hollow when faced with the lived experience of dementia. “Alzheimer’s is me unwinding, losing trust in myself… It just steals you from yourself”. This is a description by the author Pratchett (2008), who was diagnosed with dementia at the age of 60. In recent years, we have begun to hear more from those who themselves have dementia, and this has been helpful in the quest for greater understanding and empathy. Many people are affected by dementia, directly or indirectly. The idea of “unwinding” is relevant not only to the individual experience, but also to the family and community. No disease is experienced in isolation and dementia embodies this. Ideas of loss of self and loss of life feature strongly in dementia and have the potential to profoundly affect a person’s spirituality.

This paper will explore the relationship between dementia and spirituality, particularly in relation to the Christian faith. The paper will:

- address the relevance of spirituality to dementia,
- examine Psalm 71 as an example of a biblical response to ageing,
- discuss the potential effects of dementia on a person’s spirituality,
- begin to address the practical implications for carers and churches.

It is hoped that these reflections on dementia and spirituality from a Christian perspective will stimulate discussion among those of all faiths and none.

## Dementia and Spirituality

The experience of dementia asks profound questions about what it is to be human and how we are defined as people. The observed physical and psychological effects of dementia are well documented, and much effort is expended in trying to minimise the negative effects of dementia in these areas. However, to reduce people to their physical or mental characteristics alone is to neglect a huge part of human existence. The experience of living, of being the complete unit that makes up a person, is complex and multi-faceted, and there is no reason to believe that this becomes less true for those with dementia. This has often been overlooked by modern biomedicine, which is the framework for much of the discussion relating to dementia (Swinton 2001), with its emphasis on cognitive and physical problems. A potentially useful parallel is found when comparing the experience of dementia to the experience of cancer. Dame Cicely Saunders, founder of the modern hospice movement, developed the concept of “total pain”, the idea that the pain a person experiences can be a combination of physical, emotional, social and spiritual components (Baines 1990). It is possible to view dementia through a similar lens in that dementia affects every aspect of a person’s life and that the best kind of care will take these varied aspects into account.

In addition, dementia itself can be a terminal disease but is often not thought of as such (Head 2003). A retrospective study comparing acute care received by dying patients with and without dementia found that those with dementia received “fewer palliative medications or referrals to palliative care teams”. Also, less attention was paid to the spiritual needs of dying patients with dementia (Sampson et al. 2006). The study does not discuss the reasons behind these findings, but the results do show that those with dementia are treated differently. If we move beyond the bounds of the biomedical model of dementia, spirituality takes on an important role. Spirituality can be defined in a number of ways, but these definitions generally include the search for meaning, hope and transcendence. This spiritual aspect of human existence, although often intangible and taking many forms, is now recognised as a crucial aspect of being human and of importance to people with dementia (Stuckey and Gwyther 2003).

Some may question the ability to identify the spiritual needs of people with dementia – surely any needs identified are those imposed by care-givers’ perceptions, rather than the expressed needs of the individual. This may be true in the later stages of the disease process but there is often a considerable period of time after diagnosis when it is vital that discussion takes place with a person about their own concerns, fears and needs. Over the past 20 years, a growing body of literature addressing the experience of dementia has developed. Much of this consists of personal experiences from those who have dementia, including research studies, and from care-givers. A wide range of experiences is explored and spiritual concerns are included. While not everyone will write a book about their experiences, these examples show that people with dementia can and do express their feelings and experiences.

It is important to remember that the experience of dementia is different for each person. Goldsmith expresses this as a mathematical equation:

“Dementia = a person’s life history + their personality + their specific illness + their general health + their network of relationships + their ethnic identity + their physical sensory environment.” (Goldsmith 2004 adapted from Kitwood and Moore).

While spiritual concerns may be important for some people, this will not necessarily be the case for all and it would be important not to force spiritual needs on to already vulnerable people. However, it is important for care-givers to be sensitive to the existence of spiritual needs and to seek to identify and address these needs when appropriate.

## Psalm 71—A Biblical Response to Ageing

Ideas of age and ageing are common themes in the Bible. Human suffering is never denied or explained away and, consequently, the problems and challenges that commonly characterise old age are dealt with in various ways. However, it is important to remember that, for the Christian, this suffering always takes place within the context of God’s love for his people and their ultimate redemption through Christ.

Psalm 71, often described as a prayer for old age, provides a strong example of an older person giving voice to the experience of increasing age and its spiritual implications.

The author of the psalm is unknown but he appears to have had a troubled life (v7, 20). The psalmist begins by calling out to Yahweh for refuge and deliverance (v1, 2). The phrases “a rock of refuge” and “my rock and my fortress” (NRSV 1989) emphasise the steadfast nature of God and the knowledge that God is the source of deliverance. After calling out, the psalmist then moves to a remembrance of God’s presence throughout the whole length of his life. The affirmation of God’s sovereignty is important (v5) as it shows that the psalmist recognises God’s sovereignty in every aspect of his life and experience, not just when things are going well. Although initially remembering his youthful relationship with God (described by Kidner (1973) as looking back to “the limits of his memory”), the psalmist then moves beyond conventional memory to the recognition that God has been with him since birth and even before (v6). It is a powerful reminder of his lifelong relationship with God. This is of particular importance in relation to people with memory problems because it shows us that even when we are not obviously able to be consciously aware of God’s presence, in this case before birth, our lives are always in his hands. It emphasises that the relationship is of God’s devising and initiation, bringing with it resounding echoes of covenant love and grace friendship. Just as God has initiated the relationship, the psalmist can look to him to uphold it, stating “you are my strong refuge” (v7) (NRSV 1989) and this leads him to praise (v8).

Now the psalmist brings his own situation before God. In his weak and vulnerable state of old age and infirmity, there are those around him who claim that “God has forsaken” (NRSV 1989) and will not come to his aid (v11). The psalmist, reminded of God’s lifelong relationship with him, calls out “O God, do not be far from me; O my God, make haste to help me!” (v12) (NRSV 1989).

Following the affirmation of God’s grace in his life and the hope that this brings, the psalmist turns to praise (v14). He wants to proclaim God’s righteousness and salvation, even though it is too great for him to comprehend (v15). Amidst the praise, the idea of restoration is prominent. The realisation comes that the God who is with him in times of trouble, who saves him, is also the God who will bring restoration and revival (v20, 21). This eternal perspective has the potential to transform the way old age is viewed. While not minimising the many losses that increasing age can bring, the psalmist’s hope is firmly in God and the restoration he will bring—moving the focus from self to God. In this passage, we also have a reminder of the resurrection power of Christ and his victory over death (Romans 8:11). Finally, the psalmist returns to fulsome praise, highlighting the righteousness (v24) and faithfulness (v22) of God.

The idea of God’s grace and faithfulness in lifelong relationship with an individual is a key theme in the psalm. A quote from Charles Spurgeon (1867) strongly conveys this same idea. “As for His failing you, never dream of it—hate the thought. The God, who has been sufficient until now, should be trusted to the end”. The knowledge that the relationship does not depend on the individual’s intellect or ability to maintain the relationship is of particular pertinence to those with cognitive impairments and memory problems. As well as providing a bedrock for faith, it also has implications for the relationship between carer and cared-for. The importance of remembrance of God’s past works in a person’s life presents a challenge to the church and those who are carers. Those with dementia may have difficulties in accessing these memories or in expressing praise and thanksgiving. The church has a vital role to play in helping to nurture spiritual expression and experience for those of its members who cannot do so for themselves. In Romans 12, Paul instructs Christians saying, “Rejoice with those who rejoice, weep with those who weep. Live in harmony with one another; do not be haughty, but associate with the lowly; do not claim to be wiser than you are” (NRSV 1989). These words are written in the context of practical teaching about love and what it means to show God’s love in practice. In our culture, those with dementia are often placed in a very low position. The combination of increasing age and a mental health problem is imbued with a particular stigma by society. As Shamy (2003) writes “to be old…and dementing is very often to be represented as a person completely without worth”. Paul’s teaching on how to love, taken together with his teaching in 1 Corinthians 12 on the body of Christ, provide Christians with a powerful response in the face of the suffering and stigma that dementia can bring.

## Spiritual Effects of Dementia

Some of the potential effects of dementia on a person’s spirituality have already been mentioned but before we can consider specific ways to approach spiritual care for those with dementia it is necessary to consider these effects in more detail.

Swinton (2001) identifies three ways that dementia can affect the spiritual dimension:

- loss of awareness and relatedness to God/transcendence,
- loss of sense of meaning, purpose and value,
- apparent disinterest in spiritual dimension.

Swinton argues that an understanding of the experience of dementia exposes “hidden” dimensions, namely spiritual and psychological, “that are unnecessarily subsumed by the dominance of the medico-biological discourses”.

In her book, “Who will I be when I die?” Christine Boden discusses some of these issues. At the time of writing, she was 48, a mother of three children, a Christian and had previously worked at a high level in the Australian government. However, at the age of 46, she was diagnosed with early-onset Alzheimer’s disease and the book describes her life and experiences since the time of diagnosis. In the quote below, she addresses a particularly frightening aspect of dementia for her. She articulates her fear that as her illness progresses she will lose the essence of herself and that she will not be able to “hold on to her faith in God” (Boden 1998).

Here, we are given an insight into the deep spiritual questions that dementia can raise. Particularly, striking are the fears of losing faith and being alone and there are also similarities to Terry Pratchett’s description of “unwinding” and being stolen from yourself. Although these fears may be more apparent in those with a particular religious belief, it is not safe assume that only “religious” people will be affected. Questions of personhood and relationship with God and others are primarily spiritual in nature and the conclusions reached are likely to have a profound impact on the rest of a person’s life and the manner of death. Failure on the part of carers to respond to an expressed physical or emotional need would be seen as a fundamental neglect but could the same be said of expressed spiritual needs? Of course, it may not always be clear if a person with dementia is expressing spiritual need but if this aspect is not even considered then there is no possible chance of identifying it.

While working in a Christian care home for older people with dementia, I met many people who were adjusting to new circumstances and new ways of understanding the world. Some residents were Christians and had been active members of their church communities, while others had no religious affiliation and no particular interest in spirituality. One lady in particular was very articulate and interested in the world. As we spent time together, she began to share some of her concerns and worries with me. It became apparent that she had an awareness that something was wrong with her, that she could not think in the same way as before and could not rely on herself as in the past. She expressed great fear about what she was experiencing and had many questions as to the meaning and purpose of her life. In retrospect, these experiences and fears were profoundly spiritual in nature but at the time, it was difficult to know how to comfort and support this individual. To attribute her feelings to a purely physical cause, i.e. the brain pathology caused by dementia would be to rob her of her status as a human being, a unique person with unique experiences located in a community.

In seeking to understand the experiences of those with dementia, it is important to refrain from developing a “them and us” mode of thinking. The questions and fears described above could be pertinent to most us at some point in our lives. The unquestioning embracing of autonomy that so often characterises our thinking begins to be eroded in the experience of dementia. Everett (2000 cited by Goldsmith 2004) writes “People with dementia are magic mirrors where I have seen my human condition and have repudiated the commonly held societal values of power and prestige that are unreal and shallow”. Our response to this exposure of vulnerability and interconnectedness will shape our response to those with dementia. Kitwood (1997) says “dementia in another person has the power to activate fears…those concerned with dependence and frailty, and those concerned with going insane”. One response to these fears may be to marginalise those with dementia, to remove them from society. A complementary response is to “turn those who have dementia into a different species, not persons in the full sense (Kitwood 1997). For the Christian, the unique worth of being human comes from being made in the image of God, made for relationship with God and others. This applies to every human being, regardless of gender, ethnicity, intelligence, age or any other variable that exists. This can help us to see those with dementia, not as the ‘other’ or a threat, but as fully human and fully part of our communities. It becomes possible to picture a place where those with dementia are loved and valued, not because of the lives they have lived or the functions they can still perform, but because they are made in the image of the everlasting God, with the immense value that this brings.

## A Challenge to the Church

Dementia presents a challenge to the church. Many people in church communities are already affected by dementia, be that a personal diagnosis or caring for a relative. We have seen that dementia does not stop a person from bearing the image of God and, for Christians, does not disconnect them from the body of Christ. This knowledge should form the basis of the churches’ response – embodying the love of God, not forgetting those affected by dementia but embracing and ministering to them. This kind of response is described by Worthington (2007) who writes about caring for a relative with dementia. “Her personhood is held in tact by her family and other helpers. They rally together. They encircle her with love. Even as Rena’s personal identity and memory slide into oblivion due to progressive dementia, her humility is preserved in the collective memory of her loved ones.” This is the sacrificial caring that the church is called to.

However, the practical implications of this may appear daunting. Working with people with dementia is not glamorous and certainly not always easy. Goldsmith (2004) suggests that this work requires “honesty and courage” and “humility and considerable maturity”. As with other areas of ministry, it is likely that not everyone is suited or gifted for this work. Also, spiritual needs are not static, and it is to be expected that the spiritual needs of a person with dementia will change over time and through the course of the disease.

The discussion in this section will be confined to two areas: communication and participation in worship.

### Communication

Communication, in its broadest sense, is what enables relationships to be created and to grow. When communication is lost or denied people become isolated, cut off from their community. God is a god who communicates with us and who desires his children to communicate with him. While the most obvious form of this communication is found in the Bible it is by no means the only way. Psalm 19 says, “The heavens declare the glory of God; the skies proclaim the work of his hands…There is no speech or language where their voice is not heard.”

If the assumption is made that those with dementia cannot communicate or be communicated with, this aids the process of dehumanisation and allows us to forget about the person. This should not be the response of the church.

Communication can take many forms. While the ability to participate in verbal and written communication is emphasised in our culture, these are generally the types of communication that will pose the most difficulties to those with dementia. The word communicate comes from the Latin word *communicare*, meaning *to share*. Sharing can be a practical way of showing love to another. It also highlights the two-way nature of communication. It is important to be sensitive to the ways in which the person with dementia may be trying to communicate, even when this may not be obvious. Examples of non-verbal communication include music, touch and body language. Even the simple presence of another has the potential to communicate i.e. you are not forgotten, I am here with you. Other possibilities include the use of pictures that have meaning to the person with dementia. Goldsmith (2004) suggests that churches may be able to make scrapbooks with pictures of the congregation and church building. This enables people to access their past and be relocated within a loving fellowship. The principle is that communication is possible, but that it will take different forms from what may be considered the ‘normal’ (Goldsmith 1996).

### Worship

Participating in worship, whether alone or with others, is one of the ways that we identify ourselves as followers of God, actively seeking to enter into his presence. This opportunity may be denied to Christians with dementia, whether in the community or in an institution. Churches and carers may be embarrassed to have someone in the congregation who acts ‘strangely’ and so the person with dementia is not brought to church. Also, the style of a worship service may make it difficult for those with dementia to participate. This may further serve to isolate people with dementia. A number of strategies have been developed in order to aid participation in worship, notably by Shamy (2003) and Goldsmith (2004). Examples include, selecting readings and hymns likely to be familiar and careful thought to use of symbols e.g. a cross/use of a clerical collar. Worship has the potential to relocate individuals in community, to reaffirm their status as loved children, not forgotten by God and to give opportunity for praise. In a study into music therapy and spirituality for people with dementia Kirkland and Mcllveen (1999) states, “To incorporate spirituality into our own lives sometimes requires a conscious effort, regular practice, and a mindset that nurtures the human spirit.” The authors conclude that carers of those with dementia need to bring this effort and mindset to their caring practice. This is a noble aim but it should not be left to carers alone and they need to be supported in this task. It is important for churches to be aware of those of their community who have dementia and to seek to continue to minister to them, whether in church, at home or in a care home or similar institution.

## Conclusion

Dementia is a devastating disease, not least because the person appears to be lost before physical death occurs. If we focus purely on the physical and mental effects of dementia, the outlook is bleak. It has been argued that the biomedical model of dementia is insufficient and that the effect of dementia on a person’s spirituality is important. Good care should take this into account and should seek to identify and address spiritual needs. Churches need to be active in recognising these spiritual needs and in practical expression of love and ministry to those with dementia.

Psalm 88 asks “Are your wonders known in the darkness, or your righteousness in the land of forgetfulness?” (ESV 2001). The experience of dementia might lead us to ask the same of God. The exploration of Psalm 71 presents us with hope. The reassurance comes that, as Christians, our identity is held safe with God—he alone upholds us.
